# Hydrothermal Synthesis and Biocompatibility Study of Highly Crystalline Carbonated Hydroxyapatite Nanorods

**DOI:** 10.1186/s11671-015-1018-9

**Published:** 2015-08-07

**Authors:** Caibao Xue, Yingzhi Chen, Yongzhuo Huang, Peizhi Zhu

**Affiliations:** School of Chemistry and Chemical Engineering, Yangzhou University, Jiangsu, 225009 China; Shanghai Institute of Materia Medica, Chinese Academy of Sciences, Shanghai, 201203 China

**Keywords:** Hydroxyapatite, Carbonated hydroxyapatite, Nanorods, Cell viability, Alkaline phosphatase activity, MG-63 cell

## Abstract

Highly crystalline carbonated hydroxyapatite (CHA) nanorods with different carbonate contents were synthesized by a novel hydrothermal method. The crystallinity and chemical structure of synthesized nanorods were studied by Fourier transform infrared spectroscopy (FTIR), X-ray photo-electronic spectroscopy (XPS), X-ray diffraction (XRD), Raman spectroscopy, and transmission electron microscopy (TEM). The biocompatibility of synthesized CHA nanorods was evaluated by cell viability and alkaline phosphatase (ALP) activity of MG-63 cell line. The biocompatibility evaluation results show that these CHA nanorods are biologically active apatites and potentially promising bone-substitute biomaterials for orthopedic application.

## Background

Hydroxyapatite (HA) is the main inorganic component of human bone mineral, and the content of carbonate in human bone mineral is about 5–8 wt % [1, 2]. Carbonate ions in carbonated hydroxyapatites (CHA) substitute both the phosphate and hydroxyl sites of the HA structure and each is called A-type CHA and B-type CHA, respectively. Predominantly, the carbonate ions are present as B-type carbonates in natural bone minerals [3]. Synthetic CHAs have been widely used in a variety of biomedical applications including osteoconductive coatings [4–6], bone-substitute biomaterials [7], and vehicles for drug delivery [8]. Recently, hydoxyapatite nanorods have been prepared by an ethanol-induced method [9], liquid crystals [10], sonochemical synthesis [11], sol–gel method [12], and hydrothermal reaction [13, 14]. However, few methods have been reported for the preparation of carbonated hydroxyapatite nanorods with different carbonate contents. Since carbonate ion substitution in the apaptite lattice plays a major role in the biochemistry and physical properties of biological apatites, it is important to develop convenient ways for the synthesis of CHA nanorods with different carbonate contents and understand how various carbonate contents affect the crystal structure and biocompatibility of CHA nanorods.

**Table 1:** Synthesizing materials for preparing HA and CHA nanorods

Samples	Ca(NO_3_)_2_·4H_2_O/g	(NH_4_)_2_HPO_4_/g	NH_4_HCO_3_/g	EDTA/g	CTAB/g
HA	7.8870	2.6412	0	5.7000	1.0000
CHA1	7.8870	2.6412	0.2772	5.7000	1.0000
CHA2	7.8870	2.6412	0.5545	5.7000	1.0000
CHA3	7.8870	2.6412	1.1089	5.7000	1.0000

The hydrothermal method is a typical process which has been widely used in synthesis of inorganic materials for its good repeatability and crystallinity control [15–17]. In this study, we developed a hydrothermal process to synthesize carbonated hydroxyapatite nanorods with different carbonate contents, using ethylene diamine tetraacetic acid (EDTA) and cetyltrimethyl ammonium bromide (CTAB) as templates. The synthesized CHA nanorods were characterized by various analytical measurements to investigate how changes of carbonate levels affect the crystal morphology and structure of CHA nanorods. The effects of synthesized samples on the viability and osteogenic differentiation of the human osteosarcoma MG-63 cells have been measured by an MTT method and alkaline phosphate activity assay [18, 19].

## Methods

### Sample Preparation

Ca(NO_3_)_2_•4H_2_O, (NH_4_)_2_HPO_4_ and NH_4_HCO_3_ were used as a calcium source, phosphorus source, and carbonate source, respectively. Ethylene diamine tetraacetic acid (EDTA) and cetyltrimethyl ammonium bromide (CTAB) served as templates for the CHA nanorods. The phosphorus- and carbonate source solution was added dropwise to a solution of Ca(NO_3_)_2_•4H_2_O, EDTA and CTAB, meanwhile keeping pH at 9~11 by adding ammonium hydroxide solution. After 5-min stirring, the hydroxyapatite suspensions were poured into Teflon-lined stainless steel autoclaves. The autoclaves were placed in an oven for 24 h at 180 °C and then were cooled down to room temperature. The precipitate was washed by deionized water and ethyl alcohol for three times and then dried for 6 h at 80 °C. The details of synthesizing materials for preparing for HA and CHA samples are listed in Table 1.

### Transmission Electron Microscope Measurement

Transmission electron microscope (TEM, Tecnai C2 F30 S-Twin, FEI, USA) was carried out to determine particle size and morphology, and selected area electron diffraction (SEAD) was recorded by high-resolution transmission electron microscopy (HRTEM).

### Fourier Transform Infrared Spectrometry Measurement

Fourier transform infrared spectrometry (FTIR, ALPHA, Bruker, USA) was used to identify the molecular structure. After sample stage was cleaned up by ethanol wiping, the background was tested from 500 to 3600 cm^−1^. Finally, the substrate was placed on the diamond sample stage and then the cantilever was dropped onto powder slowly.

### X-ray Photo-Electronic Spectroscopy Measurement

The elements composition of the samples were analyzed by X-ray photo-electronic spectroscopy (XPS, ESCALAB250Xi, ThermoFisher Scientific, USA), using a monochromated Al Kα X-ray source.

### X-ray Diffraction Measurement

The crystalline phase of the samples was examined by X-ray diffraction (XRD, D8 ADVANCE, Bruker, Germany) with graphite monochromatized Cu Kα radiation operating at 40 kV and 40 mA at room temperature.

### Micro-Raman Spectroscopy Measurement

The molecular structure can be further analyzed by Raman spectroscopy (DXR, GX-PT-2412, Thermo, USA) with 532 nm laser as excitation wavelength. The Raman detector was equipped with a charge coupled device (CCD) multichannel detector and Olympus confocal microscope. The laser beam was focused on the sample surface and scanned for a 5-s exposure time for 180 times, meanwhile the powders were measured with extended range grating for 400–4000 cm^−1^.

### Cell Viability and Alkaline Phosphate Activity Assay Measurements

Human osteosarcoma cell line MG-63 cells were cultured in medium containing 10 % of fetal calf serum in a humidified atmosphere of 5 % CO_2_ at 37 °C, and the medium also contained 100 ug/ml streptomycin and 100 ug/ml penicillin. Then MG-63 cells were seeded in a 96-well cell culture plate with a density of 5 × 10^3^ per well. The next day, cells were treated with samples at the concentration of 0, 20, 40, 60 μg/ml. After 3 days, the cell viability was evaluated by MTT. The MG-63 cells were cultured with samples for 5 days for alkaline phosphate activity assay.

## Results and Discussion

### Morphology of CHA Nanorods

**Fig. 1:**
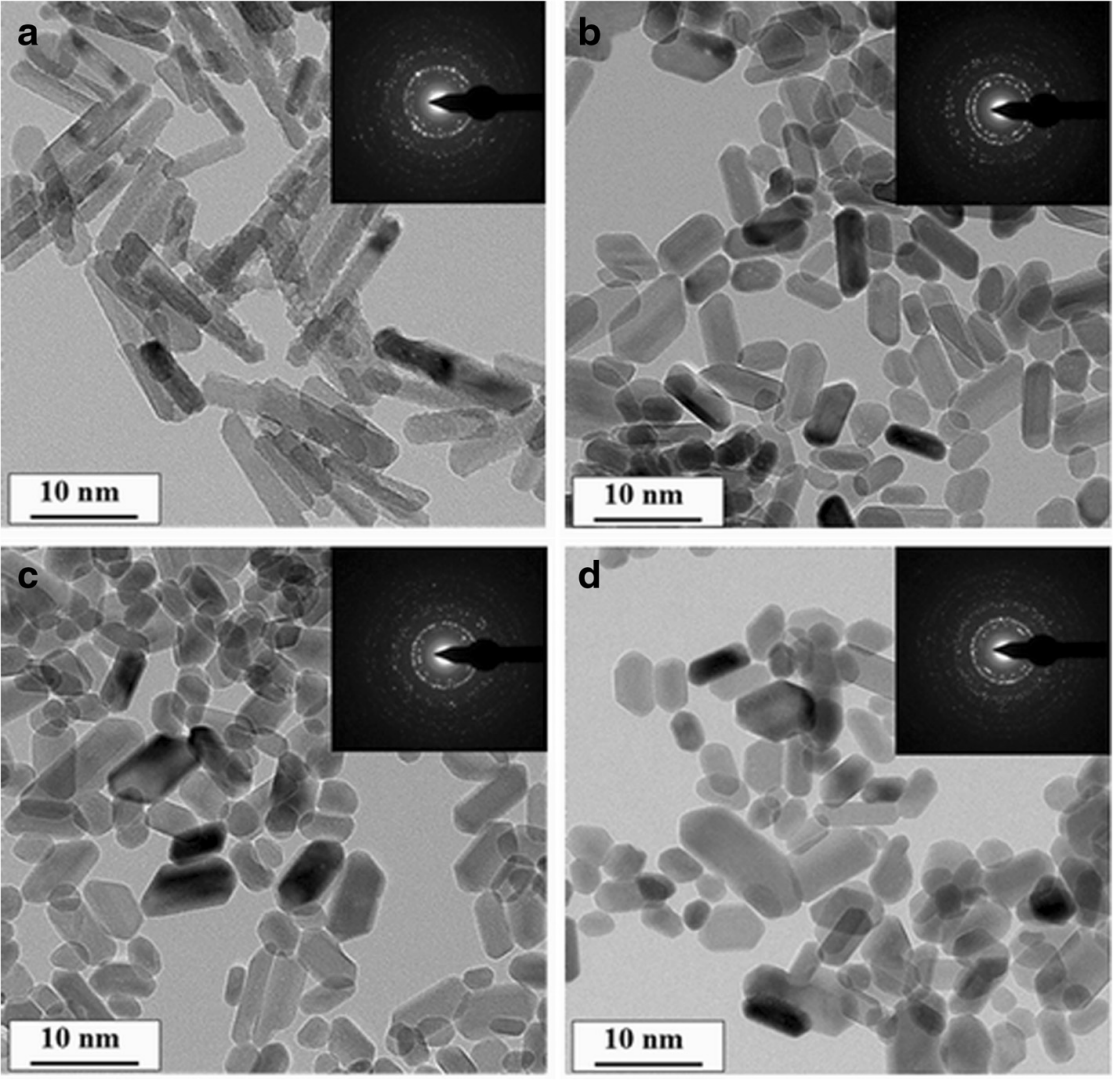
TEM image and SEAD pattern of synthesized nanorods: **a** HA; **b** CHA1; **c** CHA2; **d** CHA3

TEM was used to characterize morphology and size of synthesized nanorods. Figure 1a shows that the synthesized HA nanorods have lengths of 60–90 nm and widths of 10–20 nm, which is similar to the size of human apatite crystals [20]. As carbonate content increase (Fig. 1a–d), the lengths of nanorods decrease and the widths slightly increase. The SEAD patterns shows multi-crystalline electron diffraction concentrate rings attributed to (002), (300), (310), and (211) crystallographic planes of hydroxyapatite [21–23].

### FTIR, XPS Spectroscopy, and XRD Pattern of CHA Nanorods

**Fig. 2:**
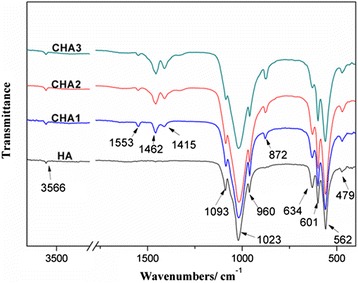
FTIR characterization of synthesized nanorods

Figure 2 shows the FTIR spectra of synthesized CHA nanorods. The broad and characteristic bands at 1023 and 562 cm^−1^ are assigned to the PO_4_^3−^ ions [24]. Three peaks at 1093, 1023, and 960 cm^−1^ should be attributed to υ_1_ and υ_3_ phosphate modes, and 601 and 562 cm^−1^ are attributed to υ_4_ phosphate modes. The antisymmetric stretching vibration of C-O (υ_3_) in the region 1500–1400 cm^−1^ indicates that different contents of CO_3_^2−^ have been doped in synthesized nanorods. The υ_2_ vibration of CO_3_^2−^ at 872 cm^−1^ and υ_3_ vibration of carbonate confirm the B-type substitution in all CHA nanorods [3].

**Fig. 3:**
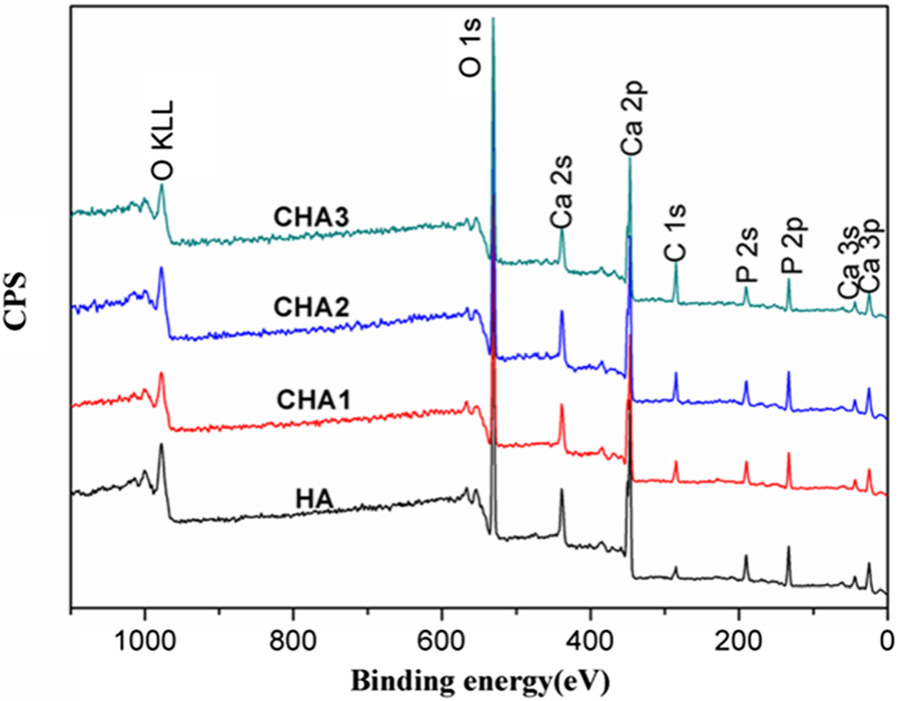
XPS characterization of synthesized nanorods

The XPS spectra of CHA nanorods containing different carbonate levels are shown in Fig. 3. One peak corresponding to C 1s was revealed at 285.1 eV, indicating that different amounts of carbonate ions have been successfully incorporated into the apatite lattice structure. The carbonate contents in HA, CHA1, CHA2, CHA3 are measured as 0.9, 1.54, 2.26, 5.22 wt %, respectively.

**Fig. 4:**
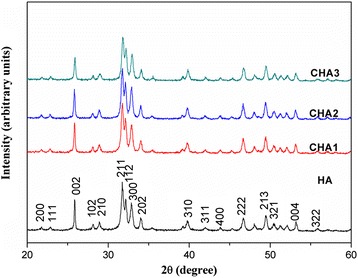
XRD patterns of synthesized nanorods

Figure 4 shows the XRD patterns of all CHA nanorods. The peaks in XRD patterns can be assigned to the (211), (112), (300), (311), (213), (004), and (002) crystallographic planes of hydroxyapatite [25]. By comparing the four XRD patterns, the diffraction peaks of CHA nanorods are a bit broader than the corresponding peaks of HA nanorod, indicating crystal lattice change induced by substitution of carbonate ions. As the carbonate content increases, the crystallinity of CHA nanorods decreases due to lattice defects caused by substitution of CO_3_^2−^ ions [26, 27].

### Raman Spectroscopy of CHA Nanorods

**Fig. 5:**
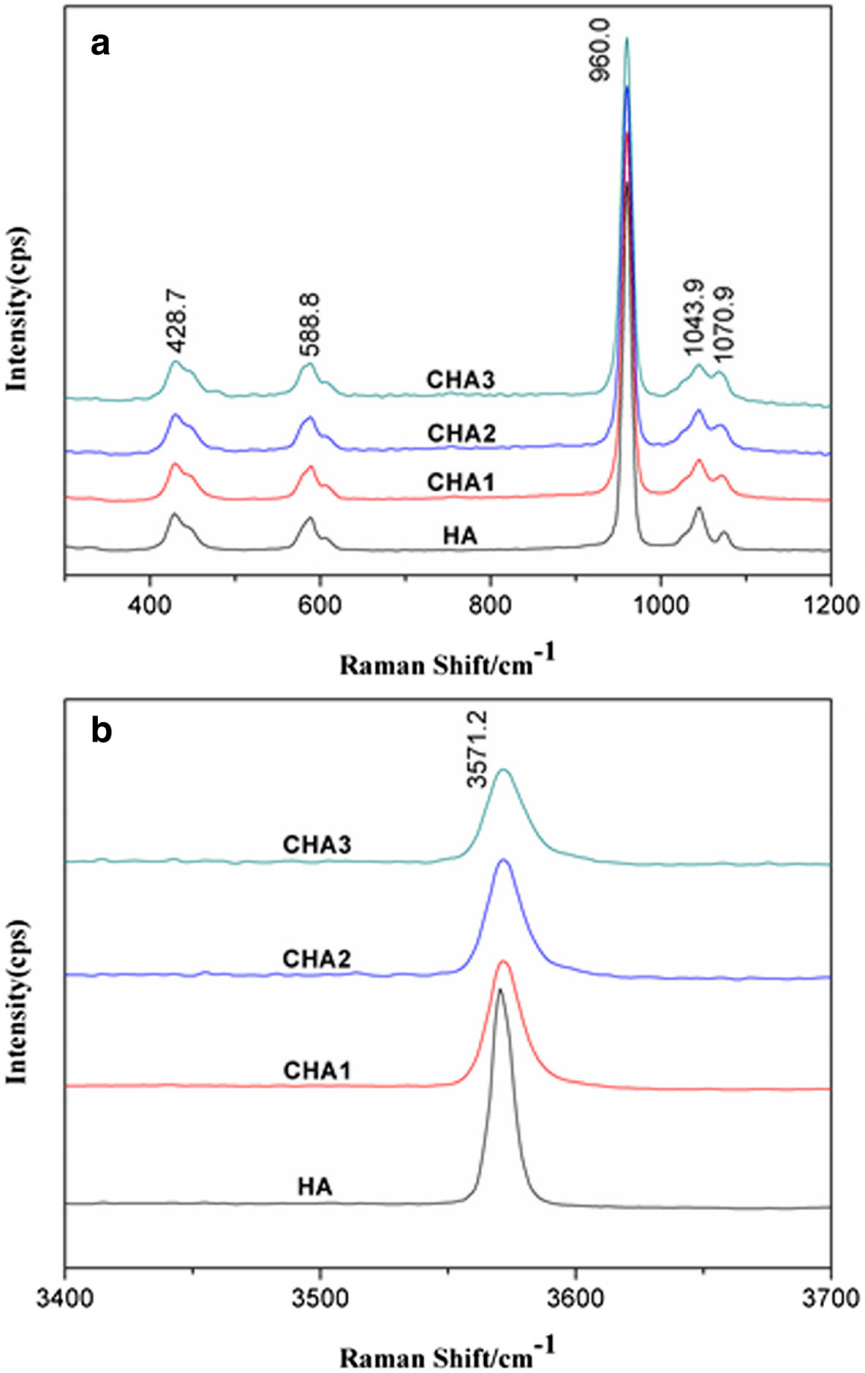
Raman spectra of synthesized nanorods: **a** in the region 300–1200 cm^-1^, **b** in the region 3400–3700 cm^-1^

Raman spectra of all nanorods are shown in Fig. 5a, b. The characteristic peaks at 428 and 588 cm^−1^ were assigned to υ_2_ and υ_4_ mode, respectively [28]. As the carbonate content increases, the strongest symmetric stretch υ_1_ mode of PO_4_^3−^ at 960 cm^−1^ becomes broader, indicating the decrease of crystallinity of apatite lattices [1, 29]. The peak at 1070 cm^−1^ can be assigned to the B-type υ_1_ CO_3_^2−^ mode [30, 31]. Figure 5b shows the decrease in intensity of the O–H stretch at about 3571 cm^−1^ (normalized to the intensity of the 960 cm^−1^ peak) with increasing carbonate content. The O–H peak position slightly shifts upfield. The substitution of PO_4_^3−^ ions by CO_3_^2−^ ions may alter the chemical environment of OH ions to cause a shift in the vibrational frequency of O–H groups. As carbonate content increases, OH peak becomes broader due to the decreasing crystallinity, which is consistent with the broadening of the 960 cm^−1^ peak.

### Cell Culture and Cell Viability Test

**Fig. 6:**
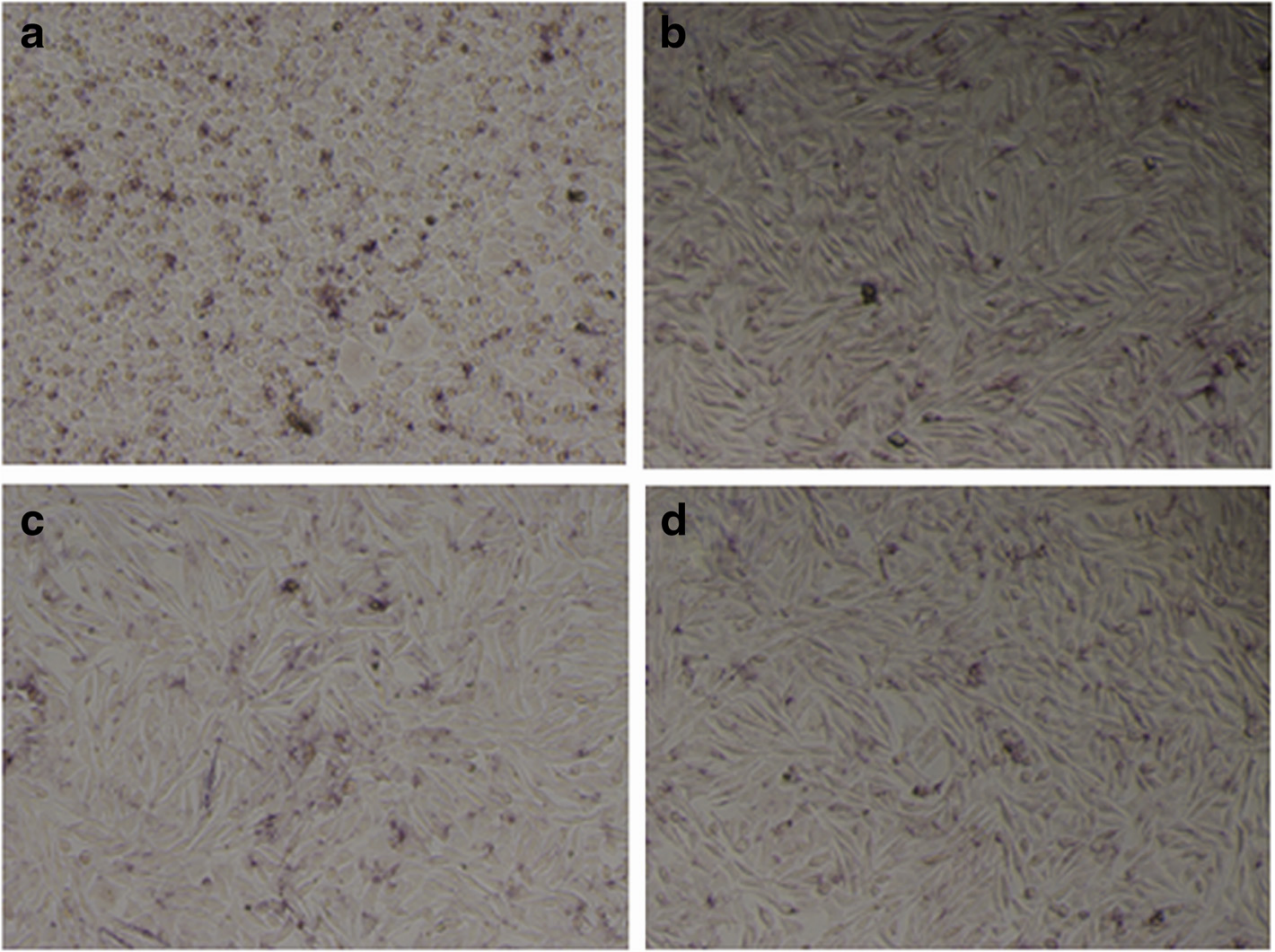
ALP activity images of MG-63 cells co-cultured with nanorods: **a** HA; **b** CHA1; **c** CHA2; **d** CHA3

As shown in Fig. 6a–d, after co-culturing for 4 days with a 60-μg/ml concentration of nanorods, alkaline phosphatase (ALP) is expressed in large amounts in the cell cytosol of MG-63 cells. Alkaline phosphatase expression is indicative of osteogenesis [14]. The ALP activity results show that all synthesized CHA nanorods with different carbonate contents had similar impacts on the growth and osteogenic differentiation MG-63 cells.

**Fig. 7:**
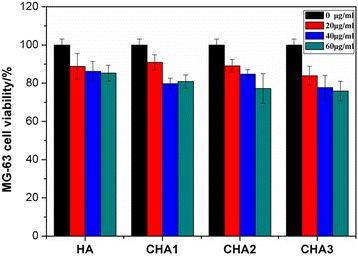
Viability of MG-63 cells co-cultured with different nanorod concentrations

The in vitro biocompatibility of CHA nanorods was also assessed by MTT assay on MG-63 cell line. The MG-63 cells were co-cultured with CHA nanorods for 3 days at the concentration of 0, 20, 40, 60 μg/ml. As shown in Fig. 7, at the concentration of 20 μg/ml, the cell viability of CHA groups was higher than or equal to the HA group. However, at the concentration of 40 and 60 μg/ml, the cell viability of all CHA nanorods is a bit lower than the HA nanorod except CHA2 at a 40-μg/ml concentration, indicating that the carbonate contents have an impact on biocompatibility of nanorods. Moreover, even at the concentration of 60 μg/ml, all cell viability was still maintained above 75 %, proving that these CHA nanorods are biological apatites and biocompatible with human osteosarcoma MG-63 cell line.

## Conclusions

We synthesized highly crystalline carbonated hydroxyapatite nanorods with different carbonate levels by a convenient hydrothermal reaction. As the carbonate content increases, the lengths of nanorods decrease, the widths of nanorods slightly increase, and the crystallinity of CHA nanorods decreases due to lattice defects caused by substitution of CO_3_^2−^ ions. The results of biocompatibility and osteogenic differentiation test prove that these CHA nanorods are biological apatites and promising biomaterials in bone-tissue engineering application.
